# Multi-species oral biofilm promotes reconstructed human gingiva epithelial barrier function

**DOI:** 10.1038/s41598-018-34390-y

**Published:** 2018-10-30

**Authors:** Lin Shang, Dongmei Deng, Jeroen Kees Buskermolen, Marleen Marga Janus, Bastiaan Philip Krom, Sanne Roffel, Taco Waaijman, Cor van Loveren, Wim Crielaard, Susan Gibbs

**Affiliations:** 1 Department of Preventive Dentistry, Academic Centre for Dentistry Amsterdam (ACTA), University of Amsterdam and Vrije Universiteit Amsterdam, Amsterdam, The Netherlands; 2 Department of Oral Cell Biology, Academic Centre for Dentistry Amsterdam (ACTA), University of Amsterdam and Vrije Universiteit Amsterdam, Amsterdam, The Netherlands; 3 Department of Molecular Cell Biology and Immunology, Amsterdam UMC, Vrije Universiteit Amsterdam, Amsterdam, The Netherlands

## Abstract

Since the oral mucosa is continuously exposed to abundant microbes, one of its most important defense features is a highly proliferative, thick, stratified epithelium. The cellular mechanisms responsible for this are still unknown. The aim of this study was to determine whether multi-species oral biofilm contribute to the extensive stratification and primed antimicrobial defense in epithelium. Two *in vitro* models were used: 3D reconstructed human gingiva (RHG) and oral bacteria representative of multi-species commensal biofilm. The organotypic RHG consists of a reconstructed stratified gingiva epithelium on a gingiva fibroblast populated hydrogel (lamina propria). Biofilm was cultured from healthy human saliva, and consists of typical commensal genera *Granulicatella* and major oral microbiota genera *Veillonella* and *Streptococcus*. Biofilm was applied topically to RHG and host–microbiome interactions were studied over 7 days. Compared to unexposed RHG, biofilm exposed RHG showed increased epithelial thickness, more organized stratification and increased keratinocyte proliferation. Furthermore biofilm exposure increased production of RHG anti-microbial proteins Elafin, HBD2 and HBD3 but not HBD1, adrenomedullin or cathelicidin LL-37. Inflammatory and antimicrobial cytokine secretion (IL-6, CXCL8, CXCL1, CCL20) showed an immediate and sustained increase. In conclusion, exposure of RHG to commensal oral biofilm actively contributes to RHG epithelial barrier function.

## Introduction

The oral mucosa of the gingiva (gum) forms a competent protective barrier between the host and its environment. Since the oral mucosa is continuously exposed to a multitude of microbes, one of its most important defense features is a thick, multi-layered and keratinized epithelium. Starting from the basal layer and migrating to the outer surface of the epithelium, epithelial cells proliferate, differentiate and are finally shed into the oral cavity, allowing for effective clearance of pathogens and rapid repair after injury^1^. The healthy oral mucosa also produces antimicrobial peptides, cytokines and chemokines which contribute to preserving the host-microbiota (multi-species biofilm) homeostasis^2–5^. The cellular mechanisms responsible for this notably high epithelial turnover and primed antimicrobial defense of the oral epithelial barrier are still unknown.

Mucosal health is based on a homeostatic balance between the tolerance of the host and colonization by microbiota. Since the oral epithelium is in close contact with the microbiota, it is possible that such interactions would have a beneficial influence on epithelial barrier properties. Indeed in the gastrointestinal system and in skin, it has been shown that some bacteria play very important roles in modulating barrier functions, such as host metabolism, tissue structure and tissue repair^6–8^. Also, gut bacteria have been shown to be necessary for immune plasticity and regulation of the adaptive immune system^6^. However, little is known about the beneficial effects of microbiota on the development of oral mucosa. One of the main reasons for this is the lack of human physiologically relevant *in vitro* models. Although conventional models combining oral cells and microbes have contributed greatly to our knowledge, they still have major shortcomings: (i) animal models do not satisfactorily represent human host-microbe interactions due to major differences in their physiology; (ii) 2D monolayer cell models do not adequately mimic the complexity of the native tissue and therefore fail to provide reliable information on morphological changes; (iii) most host-microbe interaction studies generally use mono-species, or a non-biofilm design (e.g. planktonic bacteria culture) whereas microbes *in vivo* form multi-species biofilm. This is important since a multi-species biofilm can form a microbial community with metabolic benefits which can better withstand environmental stress^9,10^; (iv) most co-culture models (conventional submerged keratinocytes and fibroblasts) are limited to 48 hours (or less) bacteria exposure^1,9,11–13^ or at the most 72 hours^14^, even though the interactions *in vivo* are in a lasting dynamic status.

In this study we combined two state of the art *in vitro* models: a 3D reconstructed human gingiva model (RHG) and multi-species oral bacteria representative of commensal biofilm, in order to study host – microbiome interactions over an extensive period of time (7 days). The organotypic RHG consists of a reconstructed stratified oral gingiva epithelium on a gingiva fibroblast populated collagen 1 hydrogel which serves as the lamina propria. Previously this RHG model has been extensively characterized and used to investigate cytokine secretion after wounding and after chemical sensitizer exposure, and also to study pathogenic biofilm immune evasion after short term (24 hr) exposure^15,16^. Whereas the RHG shows many characteristics of native gingiva epithelium (e.g. high keratin 13 and keratin 17 expression, intermittent Keratin 10 expression and extensive suprabasal involucrin expression), which distinguish it from, for example skin epidermis. However, RHG currently fails to show the highly proliferative capacity which results in the characteristic thickened epithelium compared to skin epidermis^17,18^. This suggests that extrinsic, as well as intrinsic properties of keratinocytes and fibroblasts are involved in regulating the important oral epithelium barrier properties. The aim of this study was to use the RHG model to determine whether oral microbiota contributes to oral epithelial barrier properties. The multi-species biofilm was cultured from healthy human saliva *in vitro*, and consisted of relevant numbers of bacterial species, the typical commensal genera *Granulicatella*, and predominant amounts of the major oral microbiota genera: *Veillonella and Streptococcus*^16^. This biofilm was exposed to the upper, stratified air exposed surface of RHG for 7 days and its effect on epithelial barrier properties was investigated. Epithelial stratification was assessed by quantifying the number of epithelial cell layers formed and by determining the expression of two typical proliferation markers: Ki67 and proliferating cell nuclear antigen (PCNA). Ki67 is necessary for cellular proliferation and strictly associated with active cell progression. PCNA aids DNA synthesis during DNA replication and is involved in repair after DNA damage. The expression of antimicrobial peptides (AMPs: Elafin, HBD-1, HBD-2, HBD-3, ADM and LL-37)^19,20^ and the secretion of inflammatory, antimicrobial cytokines (IL-6, CXCL8, CXCL1 and CCL20) which are known to prime the host for counteracting potential pathogens was also determined^21,22^.

## Results

### Histological features of healthy native gingiva

**Figure 1:**
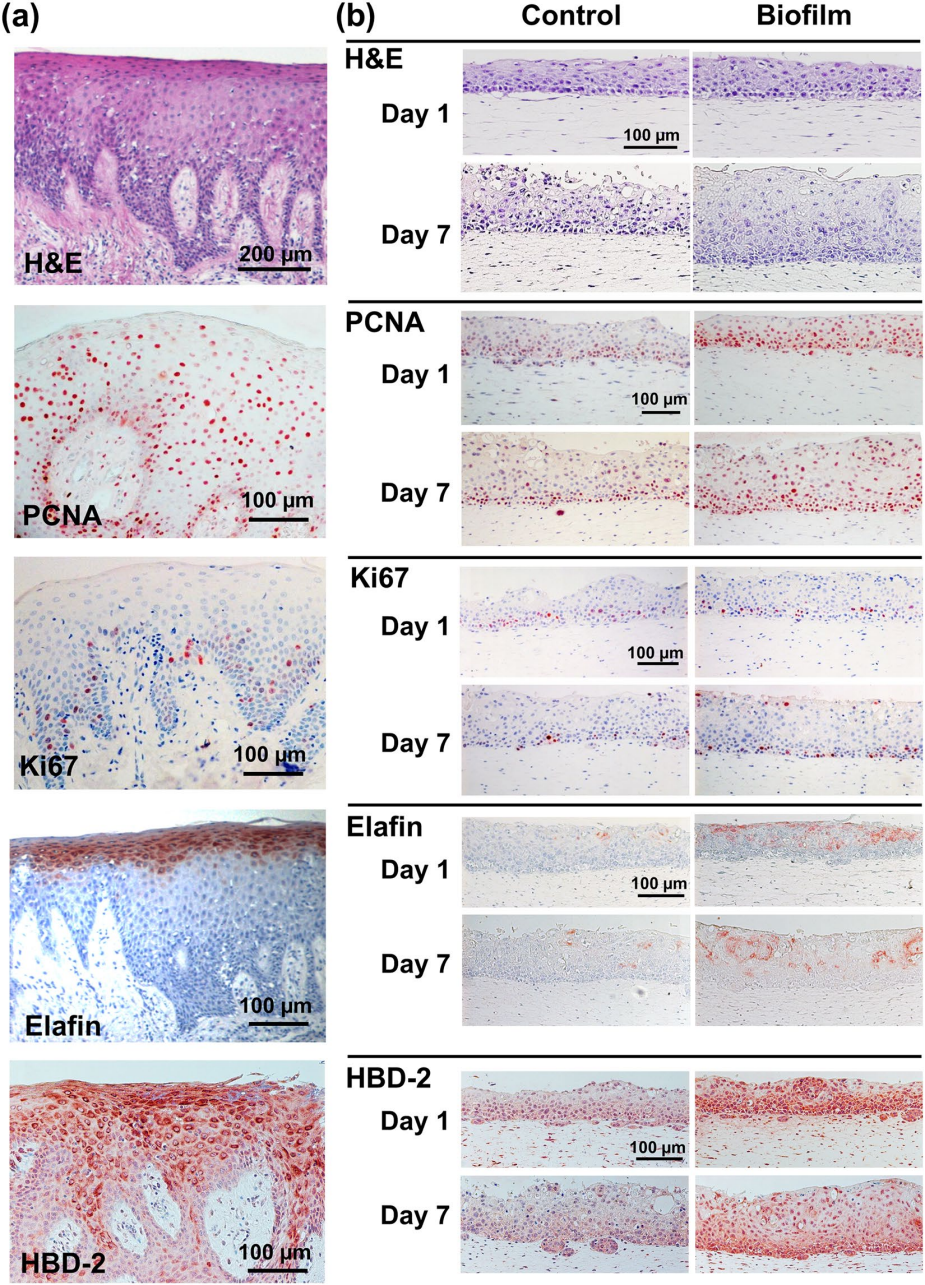
Immunohistochemical analysis of biofilm exposed RHG. (**a**) Histology of healthy native gingiva. Histology (hematoxylin and eosin staining, H&E) and immunohistochemical staining with antibodies against PCNA, Ki67, Elafin and HBD-2 is shown. (**b**) RHG were exposed topically (surface application) to either multi-species biofilm or control medium without biofilm and harvested 1 or 7 days hereafter. Histology (H & E) shows thicker epithelium at day 7 in RHG exposed to biofilm compared to control RHG. Immunohistochemistry shows increased proliferation (PCNA, Ki67), Elafin and HBD-2 in biofilm exposed RHG compared to control RHG at day 7. Figures are representative of at least three independent experiments, each with an intra-experiment duplicate.

To maintain a resistant and healthy epithelial barrier to the environment, gingiva has developed specialized morphological and functional features. The thick gingiva epithelium consists of multiple keratinocyte layers connected to the basement membrane via deep rete ridges, and forms a protective barrier above the underlying lamina propria (Fig. 1a). Rapid proliferation and self-renewal of the epithelium accelerates the clearance of toxic exogenous substances, and is indicated by two proliferation markers: PCNA and Ki67 (Fig. 1a). Abundant PCNA expression, a protein which is essential for DNA replication during cell division, was found to be expressed extensively throughout the epithelium. Ki67, a protein expressed in actively dividing cells but absent in quiescent cells, was expressed in the basal and lower suprabasal epithelial layers. The AMP elafin, a protein which inhibits serine protease and provides protection to the host^23^, was strongly expressed within the granular layer of the gingiva epithelium where host-microbe interactions begin (Fig. 1a). Another AMP, HBD-2, a protein which is expressed in normal uninflamed gingiva^24^, was also found to be abundantly express throughout gingiva epithelium (Fig. 1a).

### Multi-species biofilm increases RHG proliferation and stratification

**Figure 2:**
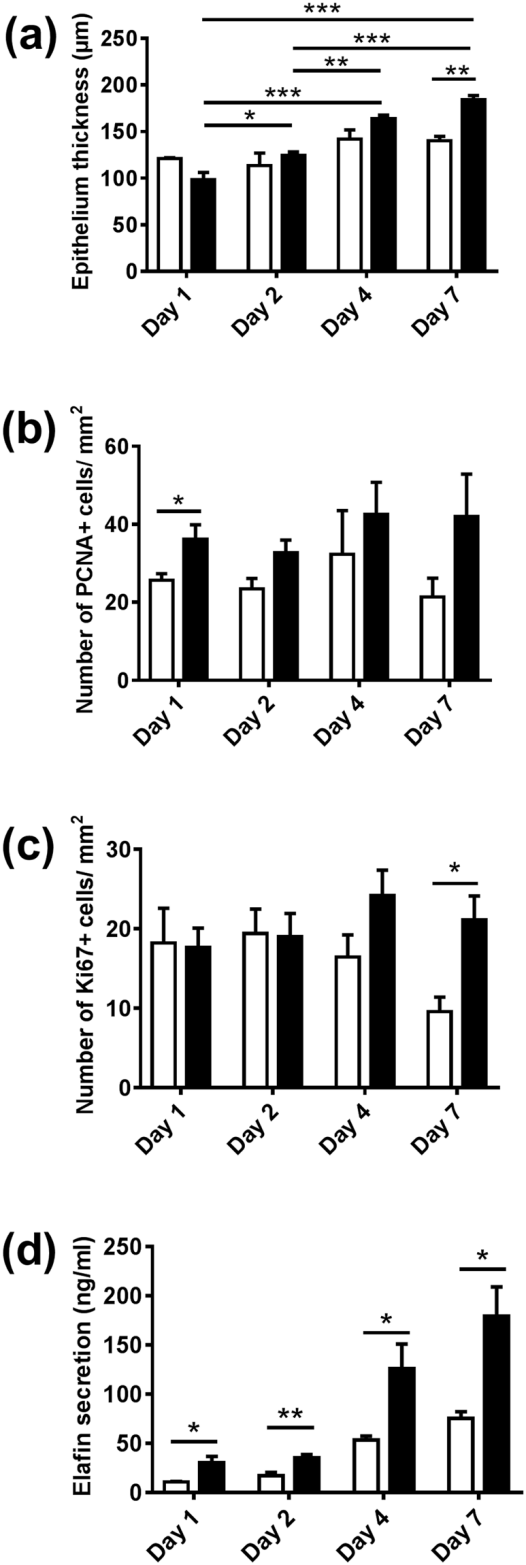
Comparison of biofilm exposed RHG with unexposed RHG over a 7 day exposure period. (**a**) Epithelial thickness, as determined from H & E stained sections, is increased in biofilm exposed RHG at day 7. (**b**,**c**) number of PCNA and Ki67 positive staining cells per mm^2^ epithelium, as determined from immune-histochemical staining of paraffin embedded tissue sections is shown: the number of PCNA-positive cells is higher in biofilm exposed RHG at day 1; biofilm exposed RHG maintain a constant number of Ki67 positive staining cells whereas control RHG senesce. (**d**) Elafin ELISA shows increased Elafin secretion into biofilm exposed RHG culture supernatant compared to control RHG. Open bar = control medium without biofilm exposed RHG; black bar = multi-species biofilm exposed RHG. Tissue samples were analyzed after 1, 2, 4 and 7 days biofilm exposure. Data represent the average of three independent experiments, each with an intra-experiment duplicate ± SEM; *p < 0.05; **p < 0.01; ***p < 0.001; unpaired *t-*test for comparison between exposed group and unexposed group and 2-way ANOVA followed by Bonferroni’s multiple comparison for comparison between time and treatment.

Since native gingiva is continuously exposed to a richly diverse microbiota, we exposed RHG to cultured oral bacteria representative of multi-species commensal biofilm and determined its effect on epithelial phenotype over a 7 day period. Notably, epithelial thickness was increased 31% in RHG exposed to biofilm compared to unexposed RHG, resulting in a similar order of thickness to that observed in native gingiva (Figs 1 and 2). This increased epithelial thickness was already apparent after a culture period of only 7 days. In the presence of the biofilm, the epithelial layers became much more organized to form a compact barrier, with a dense inner basal cell layer and with suprabasal layers becoming more differentiated (flattened anuclear keratinocytes) towards the upper surface. Due to the structure of the collagen hydrogel, rete ridges were absent both in exposed and unexposed RHG. PCNA, an early proliferative biomarker, was activated directly upon biofilm exposure (within 24 hours) resulting in a 41% increase in positively staining cell nuclei throughout the epithelium, in line with native gingiva (Figs 1 and 2). After 7 days of culture, control unexposed RHGs were senescing whereas biofilm exposed RHGs were still actively proliferating with the result that more than twice as many proliferating Ki67 positive staining nuclei were found in biofilm exposed RHGs (Figs 1b and 2). Taken together, we can conclude from these results that biofilm actively contributed to the characteristic highly proliferative stratified oral mucosa.

### Biofilm increases antimicrobial peptides and cytokine secretion in RHGs

**Figure 3:**
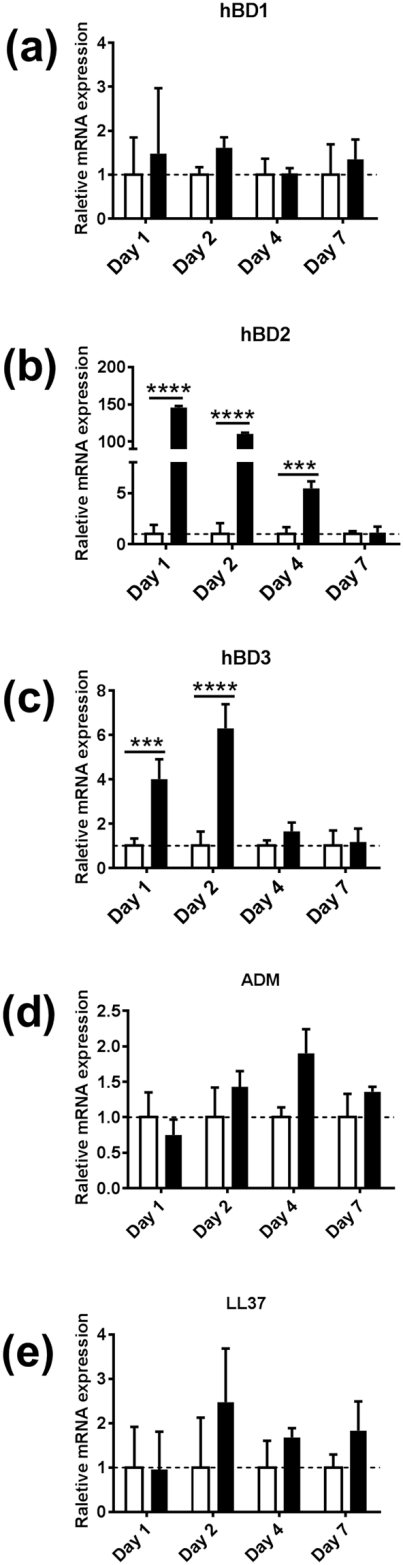
Differential response of RHG antimicrobial peptides to multi-species biofilm during a 7 day exposure period. Real time PCR analysis of mRNA transcripts for HBD1-3, ADM and LL-37. Results are shown normalized to housekeeping gene HPRT1. Open bar = control medium without biofilm exposed RHG; black bar = biofilm exposed RHG. Tissue samples were analyzed after 1, 2, 4 and 7 days biofilm exposure. Data represent the average of three independent experiments, each with an intra-experiment duplicate ± SEM; ***p < 0.001; ****p < 0.0001; unpaired t-test for comparison between exposed group and unexposed group and 2-way ANOVA followed by Bonferroni’s multiple comparison for comparison between days.

Next the influence of biofilm on the expression of epithelial antimicrobial peptides was determined. Increased amounts of Elafin were detected in the upper epithelial layers of biofilm exposed RHG, in line with Elafin location in native gingiva (Fig. 1). This was accompanied with more than 2 fold increase in Elafin secretion into culture supernatants of biofilm exposed RHG compared to unexposed RHG and this high Elafin secretion was maintained for the entire 7 day exposure period (Fig. 2d). The influence of biofilm on AMPs was further investigated on the gene expression level (Fig. 3). A clearly differential expression was observed. The expression of HBD-2 increased 145 fold already at day 1 and then gradually decreased to that of unexposed RHG at day 7 and HBD-3 expression peaked at day 2 (6.3 fold increase) and then decreased sharply to levels observed in unexposed RHG at day 4. The high increase in HBD-2 mRNA corresponded to an increase in protein expression (Fig. 1b), in line with HBD-2 expression in native gingiva (Fig. 1a). In contrast, the relative mRNA expression of HBD-1, adrenomedullin (ADM) and cathelicidins (LL-37) were not significantly increased. However, the basal mRNA expression level of these AMPs in unexposed RHG was already much higher than that of the housekeeping gene HPRT1. For example on day 1, the baseline expression of ADM was 212 fold higher than HPRT1, HBD-1 was 125 fold higher, and LL-37 was 22 fold higher (data not shown).

**Figure 4:**
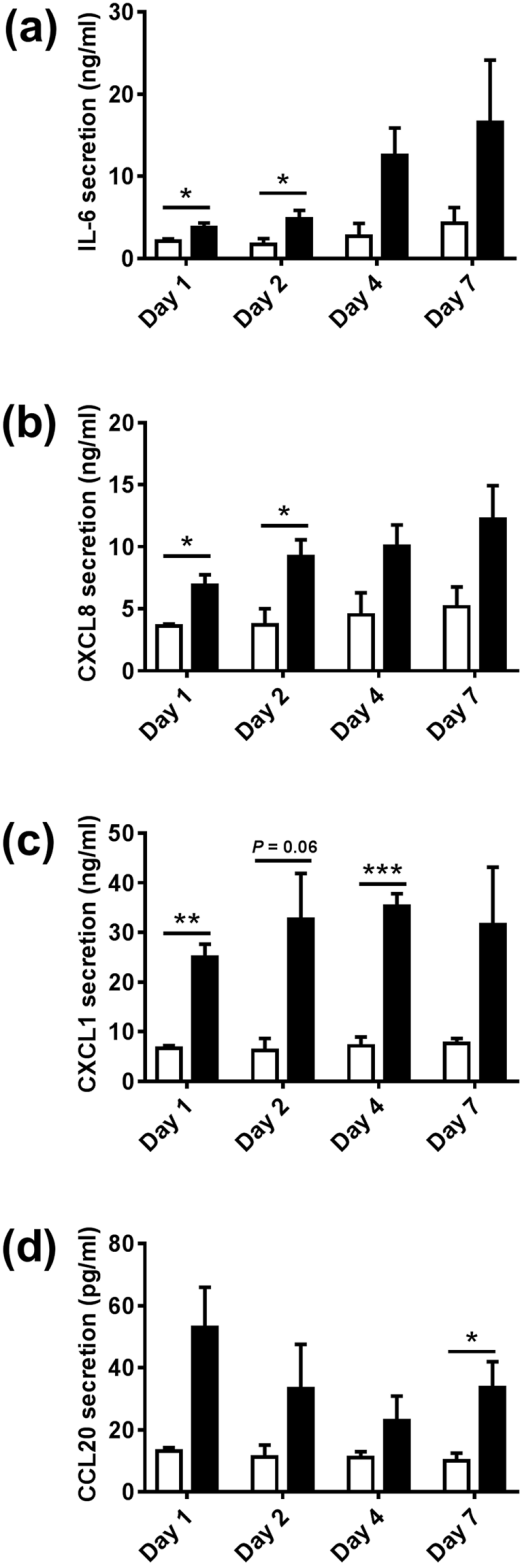
Biofilm results in a prolonged increase in cytokine and chemokine secretion from RHGs. The secretion of IL-6, CXCL8 CXCL1 and CCL20 from the lower side of RHG into culture supernatants was analyzed by ELISA. Open bar = control medium without biofilm exposed RHG; black bar = biofilm exposed RHG. Culture supernatants were analyzed after 1, 2, 4 and 7 days biofilm exposure. Data represent the average of three independent experiments, each with an intra-experiment duplicate ± SEM; *p < 0.05; **p < 0.01; ***p < 0.001; unpaired t-test for comparison between exposed group and unexposed group and 2-way ANOVA followed by Bonferroni’s multiple comparison for comparison between days.

Next the influence of biofilm on RHG inflammatory and antimicrobial cytokine secretion was determined (Fig. 4). Our results show that biofilm stimulated an immediate (within 24 hours) and prolonged (up to 7 days) secretion of IL-6, CXCL8, CXCL1 and CCL20 by RHG thus increasing RHG resistance to potential pathogens.

### Multi-species biofilm survival *in vitro*

**Figure 5:**
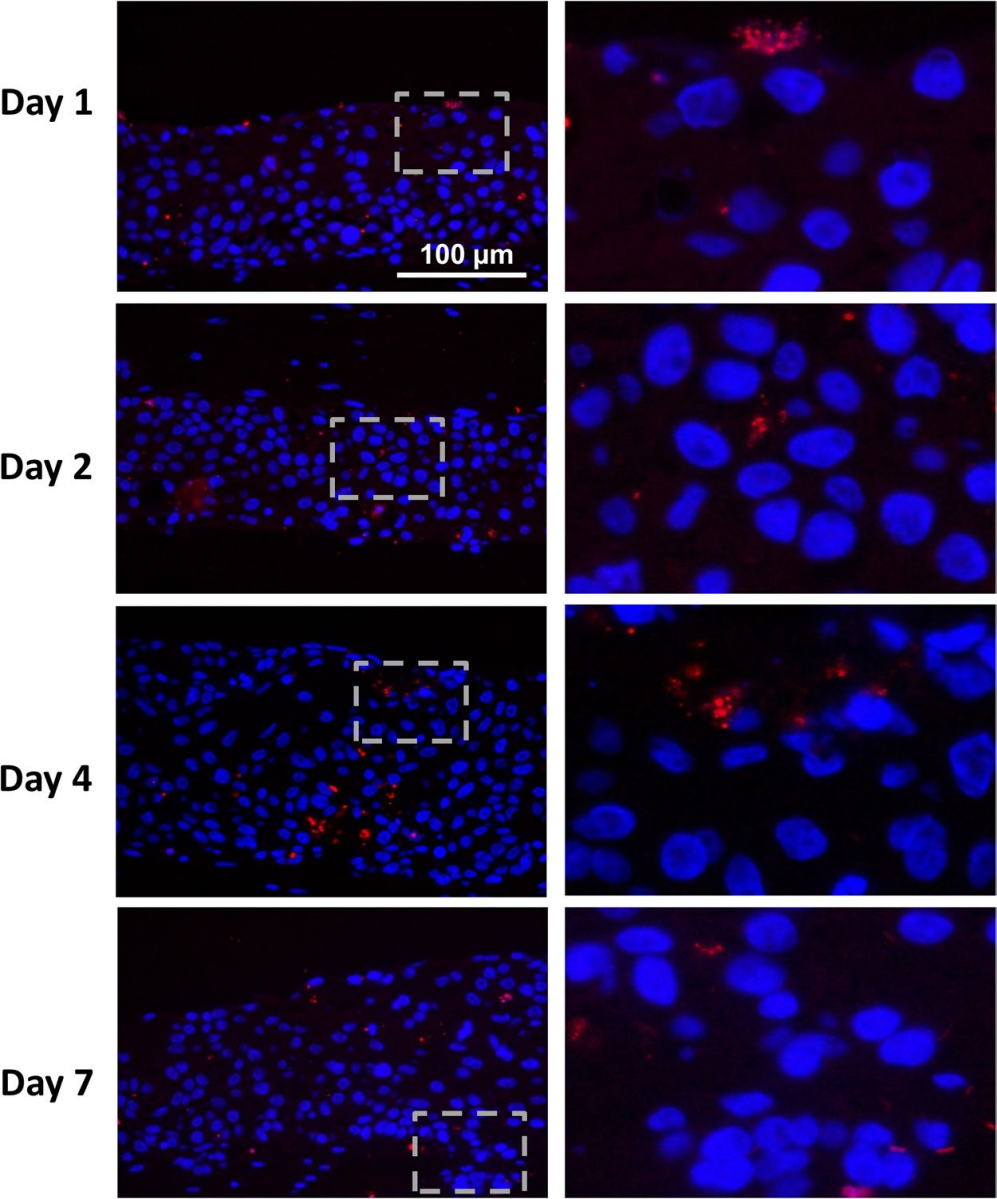
Fluorescence *in situ* hybridization (FISH) shows bacteria rRNA in the epithelium of biofilm exposed RHG. Tissue staining of paraffin embedded sections are shown. The right panel shows a higher magnification of insert shown in the left panel (dashed square). Bacteria rRNA (red) are observed distributed within the epithelium, indicating that bacteria have penetrated from the surface into the keratinocytes of the epithelial layer of RHG. Epithelial keratinocyte nuclei are stained with DAPI (Blue).

Biofilm (10^7^ CFU/ RHG sample) was applied topically to the RHG. Already after 1 day exposure, the number of CFUs which could be retrieved from the RHG was substantially less, indicating a rapid decrease in viability with time (Table 1). Notably FISH staining of bacteria rRNA was observed within the viable epithelial layers indicating that a few bacteria had penetrated to a certain extent into the tissue (Fig. 5). However, FISH staining does not distinguish live from dead bacteria at the time of RHG harvesting. Most importantly, even though viable bacteria were not detected after 2 days, the effects on the RHG were observed after 1 day and were even more pronounced after 7 days. This indicates that a single biofilm exposure, independent of whether or not the bacteria remain viable, is sufficient to stimulate long lasting effects on gingiva epithelial barrier properties in RHG.

**Table 1:** Viable bacterial cell counts of colony forming units (CFUs).

Time	Ctrl	Biofilm (CFUs/sample)^a^
Day 0^b^	ND^c^	5.47 × 10^7 ^± 1.55 × 10^7^
Day 1^d^	ND	20 ± 15
Day 2	ND	1.5 ± 2.6
Day 4	ND	ND
Day 7	ND	ND

^a^Data are represented as mean ± standard deviation, n = 6.

^b^Day 0: CFUs determined on Day 0, before applied onto RHGs.

^c^ND = not detectable, stands for counting below detection limit.

^d^Day 1–7: CFUs determined after tissue dissociation.

## Discussion

In this manuscript we show for the first time that multi-species oral biofilm has a beneficial effect on the host tissue by contributing to the unique physiological barrier properties of the epithelium found within the oral cavity. It is long known that oral mucosa has a higher turnover than for example skin and an increased thickness^18^. However the underlying mechanisms contributing to this were currently unknown. By using the organotypic RHG we were able to determine that oral bacteria representative of multi-species commensal biofilm clearly contributes to mucosa tissue integrity by increasing proliferation and stratification. Furthermore, we could show the biofilm could prime the tissue to protect against potential assault from pathogens by increasing multiple anti-microbial peptides and cytokine secretion over a prolonged period of time (7 day study period). To our knowledge, there are no studies which investigate host-microbe interactions *in vitro* for more than 72 hours and no studies which were therefore able to describe long term effects on host tissue integrity.

Our results show that exposure of RHG to the biofilm, which closely represented microbiota found in healthy saliva^25^, actively contributed to the increased epithelial thickness characteristic of oral mucosa. This finding can be explained by the observation that biofilm stimulated keratinocyte proliferation (number of Ki67 and PCNA positive cells) thus preventing the cell senescence which was observed in unexposed RHG at day 7. This would explain previous observations by us and others where sterile RHG maintained a relatively thinner epithelium with few proliferating basal keratinocytes, which was more comparable to skin epidermis than the relatively thicker gingiva epithelium^15,18,26,27^. It is possible that the moderate degree of inflammation caused by microbes is enough to stimulate an innate immune response which will result in secretion of inflammatory cytokines which also have mitogenic properties. Indeed, our results did clearly show that exposure to biofilm resulted in an immediate (within 24 hours) and prolonged (up to 7 days) inflammatory response by increasing secretion of IL-6, CXCL8, CXCL1 and CCL20. IL-6 and CXCL1 have been shown to be involved in epithelial cell proliferation and migration^21,28^.

In addition to stimulating epithelial proliferation and stratification, we found that biofilm induced an antimicrobial protective response in the gingiva epithelium, showing a selective increase in the protein expression of the Elafin, and the mRNA expressions of HBD-2 and HBD-3 but not HBD-1, ADM or LL-37. In healthy gingiva the modest level of antimicrobial peptides, cytokines and chemokines induced by biofilm may be considered to be a strategy of the host to maintain homeostasis. The host tissue, immune system and complement system will therefore be primed and kept in an activated state against potential pathogens^29,30^. Elafin, an endogenous protease inhibitor which was upregulated in our biofilm exposed RHG, plays a critical role in homeostasis by preventing tissue damage from excessive proteolytic enzyme activity during inflammation^31,32^. Healthy subjects have been reported to exhibit higher Elafin levels compared to periodontitis patients^33^. Two AMPs, HBD-2 and HBD-3, were found to be expressed constitutively in normal oral tissue in healthy people without localized inflammation^24,34^ and could be induced by resident bacteria in gingival cells *in vitro*^20,35,36^. HBD-2 was also suggested to be correlated with cellular differentiation in human gingiva epithelial cell^37^. The reason that HBD-1, ADM and LL-37 were not up-regulated in biofilm exposed RHG could be due to the absence of cell types responsible for their induction in the current RHG model. For example, LL-37 has been described to be produced by neutrophils as well as epithelial cells in oral cavity^20,24^, the former of which are not yet incorporated into our model. Alternatively, these AMPs may only be induced above base line expression in response to pathogenic biofilm rather than commensal biofilm. Our finding that biofilm resulted in increased secretion of cytokines with inflammatory as well as antimicrobial properties (IL-6, CXCL1, CXCL8 and CCL20) is in line with our previous study in which we showed that commensal biofilm stimulated a stronger innate immune response than gingivitis and cariogenic biofilm when exposed to RHG for 24 hours^16,38^. Here we show that this is a prolonged cytokine response for at least 7 days. Our *in vitro* results are in line with others who showed that indicator bacteria in healthy oral microbiota was associated with high basal levels of CXCL8 release from gingival epithelial cells obtained from healthy individuals^39^. Also, secretion of IL-6 and CXCL8 by cultured keratinocytes was shown to be promoted by less-pathogenic single-species bacteria^40^ and healthy oral microbiota^39^, but inhibited by toxic challenges e.g. live *Porphyromonas gingivalis*^40,41^.

Notably, the pronounced effect on epithelial barrier properties were observed for up to 7 days after a single biofilm exposure even though the number of viable bacteria greatly decreased within the first day of exposure. This would indicate that a single bacterial trigger is enough to result in a long lasting effect or that dead bacteria on the upper surface of the epithelium are still able to trigger a response. There is accumulating evidence which suggests that host-microbiome responses are associated with cellular signaling pathways such as the mitogen-activated protein kinase (MAPK) and Toll-like receptor pathways. These in turn are widely involved in the regulation of cellular proliferation, differentiation, and immune response to inflammation^2,29^. Evidence suggests that lipopolysaccharide (a molecule present on the bacteria cell wall) is sufficient to trigger MAPK and Toll-like receptor pathways which would suggest that viable bacteria are not required^42,43^. However, this needs further investigation as to whether lipopolysaccharide alone would result in our observed epithelial phenotypic changes.

The limitations of our study should also be noted. The biofilm was cultured from pooled healthy saliva in such a way that it maintained similar phenotypic features to the *in vivo* oral microbiota^16^. However due to the methodology used to create enough biofilm to expose RHG in a reproducible manner, the intact structure of the preformed biofilm was inevitably disrupted. The most probable explanation for the loss of viability of the biofilm is that RHG were cultured under aerobic conditions whereas the biofilm used in these experiments prefers anaerobic culture conditions. Thus applying biofilm to the surface of RHG followed by culturing under aerobic conditions would be expected to result in the observed decrease in CFUs. Although challenging, in the future anaerobic biofilm conditions should be optimized for RHG aerobic exposure. Alternatively, the antimicrobial responses elicited in the RHGs, which showed significantly higher levels than in unexposed RHG, may have contributed to the decrease in viability of the biofilm. Our results could also possibly be explained in part by a selective sub-set of survivors derived from the original biofilm. However, it is beyond the scope of this manuscript to isolate and characterize the FISH positive invading bacteria. Furthermore, FISH (bacteria rRNA staining) does not guarantee viable bacteria in the epithelium since DNA can be isolated from dead bacteria. Another limitation in our study is the lack of immune cells. However we consider it important to introduce complexity where complexity is required. In this present study we aimed to determine whether biofilm had a beneficial influence on oral mucosa tissue integrity, and in particular directly on the epithelium. Therefore the experimental design was kept relatively simple. Indeed in future studies we will introduce Langerhans Cells in a similar manner to our skin models as Langerhans Cells are key antigen presenting cells in sampling pathogens^44^. Furthermore, additional cell types such as monocytes and neutrophils will be added in order to further compare commensal and pathogen host responses.

In conclusion, we show that in the presence of the biofilm, RHG developed both morphological and functional features similar to those of native gingiva, indicating that the healthy multi-species biofilm, to a certain extent, promotes a positive symbiosis in the host. Our results highlight the contribution of multi-species biofilm in promoting gingiva epithelium barrier integrity *in vitro*, therefore providing new insights and possibilities for studying host-microbe interactions.

## Methods

### Healthy native gingiva

Healthy human gingiva tissue was obtained after informed consent from patients undergoing wisdom tooth extraction as previously described^45^. The tissue was used in an anonymous fashion in accordance with the “Code for Proper Use of Human Tissues” as formulated by the Dutch Federation of Medical Scientific Organizations (www.fmwv.nl) and following procedures approved by the institutional review board of the VU University Medical Centre.

### Culture of RHGs and multi-species biofilm

RHG: the immortalized human gingiva keratinocyte (KC-TERT, OKG4/bmi1/TERT, Rheinwald laboratory, Boston, MA, USA^46,47^) and fibroblast (Fib-TERT, T0026, ABM, Richmond, BC, Canada) cell lines were used to construct RHG as described previously^15^. RHG were cultured at the air – liquid interface and were composed of a differentiated epithelium on a fibroblast-populated collagen hydrogel. Culture medium (DMEM/Ham’s F12 (3/1) (Gibco, Grand Island, USA) supplemented with 1% Fetal Clone III (RHG, Logan, UT, USA), 1% penicillin–streptomycin (Gibco), 0.1 µM insulin (Sigma-Aldrich, St. Louis, MO, USA), 2 µM hydrocortisone (Sigma-Aldrich), 1 µM isoproterenol (Sigma-Aldrich), 10 µM carnitine (Sigma-Aldrich), 10 mM L-serine (Sigma-Aldrich), 0.4 mM L-ascorbic acid (Sigma-Aldrich), and 2 ng/mL epidermal growth factor (Sigma-Aldrich).

Oral bacteria representative of multi-species commensal biofilm: pooled human saliva from 10 healthy donors was used as inoculum for multi-species biofilm as previously described^25^. The 10 donors were considered healthy since they had no complaints which required treatment by a dental specialist. The saliva was collected following the ethical principles of the 64th World Medical Association Declaration of Helsinki and the procedures approved by the institutional review board of the VU University Medical Centre (Amsterdam, The Netherlands). Informed consent was obtained from all participants. The biofilms were formed in the Amsterdam active attachment model (AAA-model^48^). The anaerobic colony forming units (CFU) of the biofilm were assessed as a measure of viable bacterial cell counts before use. Aliquots were frozen at −80 °C until use.

Biofilm application to RHG: The stored biofilm was thawed on ice, centrifuged and dispersed in Hanks’ balanced salt solution (Sigma-Aldrich)^49^. A sample of biofilm was processed to determine CFU at Day 0. The remaining biofilm was used to apply to the upper surface of RHG as follows: RHG were topically exposed to approximately 1 × 10^7^ CFU biofilm cells concentrated in a drop of 10 µl, further cultured at the air-liquid interface at 37 °C, 7.5% CO_2_ and 95% humidity and harvested 1, 2, 4, or 7 days hereafter. The RHGs were divided into two halves for conventional paraffin embedment or determination of CFUs.

### Histology and Fluorescence *In Situ* Hybridization

After paraffin embedment, tissue sections (5 µm) were stained with hematoxylin and eosin (Histology; H&E), or processed for immunohistochemistry to determine expression of PCNA (Dako, Santa Clara, CA, USA), Ki67 (Dako), Elafin (TRAB2O, Hyult Biotech, The Netherlands) or HBD-2 (OriGene Technologies, Rockville, USA) as described previously^16,50^. For PCNA and Ki67 labeling, positive cells were quantified from at least 8 images obtained from 3 independent experiments under a magnification of 20x.

FISH rRNA *in situ* hybridization was performed on paraffin sections according to the FISH kit instructions (10MEH000; Ribo Technologies, Groningen, The Netherlands) with the probe EUB338 (5’ – GCTGCCTCCCGTAGGAGT). After the staining procedure, the sections were counterstained with DAPI and sealed using a mounting reagent (Fluoroshield, Abcam, Cambridge, UK).

### ELISA

Culture supernatants from RHG were collected at the time of harvesting and used to detect levels of IL-6, CXCX1, CXCL8, CCL20 and Elafin using enzyme-linked immunosorbent assays (ELISAs). With the exception of CXCL8 (Sanquin, Amsterdam, The Netherlands) and Elafin (PI3 Human ELISA Kit, Thermo Fisher Scientific, Maryland, USA) where ELISA kits were used, antibodies and recombinant proteins were purchased from R&D Systems, Inc. (Mineapolis, USA) and ELISAs performed according to recommendations of the supplier.

### Real-time RT-PCR

RHG epithelium was removed from the collagen hydrogel, lysed and total RNA was isolated using a RNA/Protein preparation kit (Qiagen, Hilden, Germany). Genomic DNA elimination and cDNA synthesis were performed using a reverse transcription kit (Qiagen) following the manufacturer’s guideline. Real-time PCR was performed using RT^2^ SYBR® Green qPCR Mastermixes (Qiagen) with paired-primers for human beta defensin 1-3 (HBD 1-3; HP208395, HP208178, HP213186), adrenomedullin (ADM; HP205068), cathelicidin antimicrobial peptide (CAMP; HP207673) or housekeeping gene HPRT1 (HP200179), all purchased from OriGene Technologies, Rockville, USA. Briefly, 2 µl cDNA was added to 1 µl paired-primer, 9.5 µl nuclease-free water and 12.5 µl of SYBR green mastermix. The cycle threshold value was defined as the number of PCR cycles where the fluorescence signal exceeds the detection threshold value. Normalized by the expression of housekeeping gene HPRT1, the targeted mRNA induction was calculated by the ∆∆C_T_ analysis method following the formula:

$${2}^{-{\rm{\Delta }}{\rm{\Delta }}{\rm{C}}{\rm{T}}}=\frac{{2}^{-[\text{CT}({\rm{t}}{\rm{a}}{\rm{r}}{\rm{g}}{\rm{e}}{\rm{t}})-\text{CT}({\rm{H}}{\rm{P}}{\rm{R}}{\rm{T}}1)]}\,{\rm{e}}{\rm{x}}{\rm{p}}{\rm{r}}{\rm{e}}{\rm{s}}{\rm{s}}{\rm{i}}{\rm{o}}{\rm{n}}}{{2}^{-[\text{CT}({\rm{t}}{\rm{a}}{\rm{r}}{\rm{g}}{\rm{e}}{\rm{t}})-{\rm{C}}{\rm{T}}({\rm{H}}{\rm{P}}{\rm{R}}{\rm{T}}1)]}\,{\rm{c}}{\rm{o}}{\rm{n}}{\rm{t}}{\rm{r}}{\rm{o}}{\rm{l}}}$$

### Viable bacterial cell counts

To determine the biofilm viability at different co-culture time points, total CFUs were counted. For Day 0, serial dilutions of the dispersed biofilm were made and plated on tryptic soy blood agar plates. For Days 1–7, RHG were dissociated using a tissue dissociator (gentleMACS, Miltenyi Biotec B.V., The Netherlands), followed by sonication and plating on tryptic soy blood agar plates. The plates were subsequently incubated anaerobically for 7 days at 37 °C and the CFUs were counted.

### Statistics

Statistical analysis was performed with SPSS Statistics (version 23). RHG data were collected from at least three individual experiments, each with an intra-experiment duplicate. The thickness, PCNA, Ki67, Elafin, cytokine secretion were analyzed using an unpaired *t-*test (between exposed RHG and unexposed RHG on Day 1, 2, 4 and 7). Comparison between time and treatment of the thickness, PCNA, Ki67, Elafin, cytokine secretion and mRNA expression were analyzed using 2-way ANOVA followed by Bonferroni’s multiple comparison. Differences were considered significant when *p* < 0.05. Data are represented as mean ± standard error of mean; *p < 0.05; **p < 0.01; ***p < 0.001; ****p < 0.0001.

## Data Availability

The datasets generated during and/or analyzed during the current study are available from the corresponding author on reasonable request.
